# Molecular Characteristics, Oncogenic Roles, and Relevant Immune and Pharmacogenomic Features of EVA1B in Colorectal Cancer

**DOI:** 10.3389/fimmu.2022.809837

**Published:** 2022-02-16

**Authors:** Bin Ma, Kangchun Wang, Yu Liang, Qingkai Meng, Yongmin Li

**Affiliations:** ^1^Department of Colorectal Surgery, Cancer Hospital of China Medical University, Liaoning Cancer Hospital and Institute, Shenyang, China; ^2^Department of Organ Transplantation and Hepatobiliary, the First Affiliated Hospital of China Medical University, Shenyang, China

**Keywords:** colorectal cancer, EVA1B, prognosis, tumor immunity, pharmacogenomic features

## Abstract

**Objective:**

EVA1B, a protein coding gene, is a critical paralog of EVA1A gene. Herein, our study was conducted to investigate the role of EVA1B in colorectal cancer (CRC) progression and prognosis.

**Methods:**

Pan-cancer analysis was conducted to analyze expression, genetic and epigenetic alterations, and immunological characteristics of EVA1B. Especially, immunological characteristics and mutational landscape were compared between high and low EVA1B expression groups in the combined TCGA-COAD and TCGA-READ datasets. Through random survival forest analysis, an EVA1B-derived genomic model was developed, and its prognostic value was verified in the external datasets (GSE14333, GSE39582, and GSE87211). Drug sensitivity was compared between high- and low-risk subpopulations. A nomogram was conducted through integrating independent factors.

**Results:**

EVA1B expression presented a remarkable upregulation in most cancer types, especially CRC. EVA1B expression was significantly correlated to DNA methyltransferases, DNA mismatch repair genes, m^6^A regulators, TMB, and MSI across pan-cancer. High EVA1B expression indicated an undesirable CRC patients’ prognosis. Additionally, its upregulation was correlated to enhanced immune cell infiltration, increased stromal and immune activation, and elevated activities of cancer immunity cycle. Higher frequencies of amplification and deletion were investigated in high EVA1B expression subpopulation. Following verification, the EVA1B-derived genomic model reliably predicted patients’ prognosis and drug responses. The nomogram (age, stage, EVA1B-derived risk score) was conducted to quantify an individual’s survival probability. Furthermore, our experimental validation based on immunohistochemistry indicated that EVA1B overexpression is correlated with CRC tumorigenesis and poor outcomes in our CRC patients’ cohort.

**Conclusion:**

Collectively, our findings provided valuable resource for guiding the mechanisms and therapeutic analysis of EVA1B in CRC.

## Introduction

Colorectal cancer (CRC) involving the colon and rectum ranks the third leading cause of cancer mortality globally, with over 1.85 million cases and 850,000 deaths each year (1). Recently, CRC presents increasing incidence among younger cases (2). This disease is a heterogeneous disease with distinct pathogenesis mechanisms, involving somatic mutation, genetic fusion, genetic instability, as well as epigenetic alteration (3). Among newly diagnosed CRC, 20% of cases have occurring metastasis at diagnosis as well as 25% will metastasize following localized disease (1). Surgical resection is the major therapeutic regimen of CRC. Nevertheless, there are very few treatment regimens for metastatic patients. Although chemotherapy is usually recommended, merely few targeted therapies appropriate for cases who have specific mutation profiling are available, such as EGFR inhibitor and VEGF inhibitor (3). Hence, it is urgently required to develop novel molecule targets against CRC.

Immunotherapy is a novel alternative against CRC treatment, which utilizes cases’ own immune system to combat tumor cells (4, 5). A minority of CRC cases present microsatellite instability (MSI), a molecule predictor of defective DNA mismatch repair, and a predictive biomarker of immunotherapeutic response, but most of them are microsatellite-stable (MSS) (6). Enhanced tumor mutational burden (TMB) and neoantigen load in MSI cancers sustain the infiltrations of immune effector subpopulations, and anti-cancer immune response is strongly in relation to MSS counterparts (6). Nevertheless, a few MSS cancers present increased TMB as well as infiltrating immune cells to respond to immunotherapy. Hence, novel predictors of immunotherapeutic responses will be required. EVA1B is a protein coding gene, which is a critical paralog of EVA1A gene. It is an endoplasmic reticulum and lysosome-relevant protein involving autophagy and apoptosis (7). Previously, it triggers papillary thyroid carcinogenesis and epithelial-mesenchymal transition (EMT) through Hippo signaling (8). Flubendazole exerts an antitumor role through modulating EVA1A-mediated autophagy and apoptosis in breast carcinoma (9). MiR-125b relieves oxaliplatin resistance in liver carcinoma through negatively modulating EVA1A-mediated autophagy (10). EVA1B possesses high sequence similar to EVA1A gene, and EVA1B protein presents the similar domain to EVA1A protein (11). Nevertheless, the role of EVA1B in CRC remains indistinct. This study was conducted to investigate the correlation of EVA1B expression with prognosis, tumor immune and pharmacogenomic features in CRC.

## Materials and Methods

### Pan-Cancer Analyses

RNA sequencing data (FPKM), somatic mutational data [Mutation Annotation Format (MAF)], copy number variation (CNV) data, and clinical information of 33 cancer types [adrenocortical carcinoma (ACC); bladder urothelial carcinoma (BLCA); breast invasive carcinoma (BRCA); cervical squamous cell carcinoma and endocervical adenocarcinoma (CESC); cholangiocarcinoma (CHOL); colon adenocarcinoma (COAD); lymphoid neoplasm diffuse large B-cell lymphoma (DLBC); esophageal carcinoma (ESCA); glioblastoma multiforme (GBM); head and neck squamous cell carcinoma (HNSC); kidney chromophobe (KICH); kidney renal clear cell carcinoma (KIRC); kidney renal papillary cell carcinoma (KIRP); acute myeloid leukemia (LAML); lower-grade glioma (LGG); liver hepatocellular carcinoma (LIHC); lung adenocarcinoma (LUAD); lung squamous cell carcinoma (LUSC); mesothelioma (MESO); ovarian serous cystadenocarcinoma (OV); pancreatic adenocarcinoma (PAAD); pheochromocytoma and paraganglioma (PCPG); prostate adenocarcinoma (PRAD); rectum adenocarcinoma (READ); sarcoma (SARC); skin cutaneous melanoma (SKCM); stomach adenocarcinoma (STAD); testicular germ cell tumors (TGCT); thyroid carcinoma (THCA); thymoma (THYM); uterine corpus endometrial carcinoma (UCEC); uterine carcinosarcoma (UCS); uveal melanoma (UVM)] were curated from The Cancer Genome Atlas (TCGA) through the GDC data portal (https://portal.gdc.cancer.gov/). Tumor Immune Estimation Resource (TIMER; version 2.0) web tool (http://timer.cistrome.org/) was adopted to analyze the expression of EVA1B across 33 cancer types (12). For TCGA dataset, FPKM value was transformed to TPM value. The expression profiling of four major DNA methyltransferases (DNMT1, DNMT2, DNMT3A, and DNMT3B), DNA mismatch repair genes (MLH1, MSH2, MSH6, PMS2, and EPCAM), and m^6^A regulators (CBLL1, VIRMA, METTL3/14, RBM15/15B, WTAP, ZC3H13, ALKBH5, FTO, ELAVL1, FMR1, HNRNPA2B1, HNRNPC, IGF2BP1/2/3, LRPPRC, YTHDC1/2, and YTHDF1/2/3) was extracted from pan-cancer specimens. TMB and MSI of pan-cancer were also harvested from TCGA project. Spearman correlation analysis was conducted to investigate the correlation of EVA1B with above factors across pan-cancer.

### Estimation of Immunological Features

The abundance of tumor-infiltrating immune cells was inferred with single-sample gene set enrichment analysis (ssGSEA) derived from gene set variation analysis (GSVA) package (13). In total, 122 immunomodulatory factors containing MHCs, receptors, chemokines, and immune stimulators were collected from Charoentong et al. (14). Additionally, immune checkpoint molecules were retrieved from the study of Auslander et al. (15). Overall infiltration of stromal and immune cells was estimated in CRC tissues with estimation of stromal and immune cells in malignant tumors using expression data (ESTIMATE) algorithm in accordance with mRNA expression data (16). Cancer immunity cycle was curated from previous research (17), and the activities of all steps were estimated with ssGSEA (18).

### Collection of CRC Datasets

The GSE14333 (19), GSE39582 (20), and GSE87211 (21) datasets were collected from the Gene Expression Omnibus (GEO; https://www.ncbi.nlm.nih.gov/geo/) project. Batch effects were adjusted with ComBat algorithm derived from sva package (22). Somatic mutational data were visualized with maftools package (23). GISTIC2.0 was adopted to analyze amplification and deletion using CNV data (24). Raw “CEL” files of microarray profiling from Affymetrix platform were downloaded, followed by background correction and quantile normalization *via* robust multiarray averaging algorithm utilizing affy and simpleaffy packages (25, 26). Meanwhile, normalized matrix files of microarray profiling from other platforms were directly curated.

### Curation of Gene Sets of Known Biological Processes

Gene sets of known biological processes containing CD8+ T effector, DNA damage repair, pan-fibroblast TGF-β response signature (pan-F-TBRS), antigen processing machinery, immune checkpoints, EMT1-3, FGFR3-relevant gene signatures, angiogenesis, KEGG discovered histones, Fanconi anemia, cell cycle, DNA replication, nucleotide excision repair, homologous recombination, mismatch repair, WNT target, as well as cell cycle regulators were curated from previous research (27–29). The activities of biological processes were quantified with ssGSEA algorithm.

### Functional Enrichment Analysis

GSEA was conducted to investigate the differences in signaling pathways activated in two subpopulations, with the gene set “c2.cp.kegg.v6.2.symbols.gmt” as the reference. Gene ontology (GO) and Kyoto Encyclopedia of Genes and Genomes (KEGG) pathway enrichment analysis of EVA1B-relevant genes was conducted with clusterProfiler package (30). GO categories contained biological process (BP), cellular component (CC), and molecular function (MF). Hallmark gene sets were harvested from the Molecular Signatures Database (31), and their activities were quantified with ssGSEA algorithm.

### Screening EVA1B-Relevant Genes

To identify EVA1B-relevant genes, CRC patients were classified into high and low EVA1B expression subpopulations with the mean value of EVA1B expression. Through empirical Bayesian method from limma package (32), differentially expressed genes between subpopulations were determined. The significance criteria of EVA1B-relevant genes were set as |fold-change| >1.5 and adjusted *p*-value <0.01.

### Exploitation of an EVA1B-Derived Genomic Model

Through univariate Cox regression models, interaction of EVA1B-relevant genes with CRC prognosis was estimated in TCGA cohort. EVA1B-relevant genes with *p*-value <0.05 were determined as prognostic factors, which were input into random survival forest analysis (33). The number of Monte Carlo iterations was set as 100, and the number of steps forward was 5. Thereafter, their relative importance was ranked. In accordance with relative importance >0.28, characteristic EVA1B-relevant genes were determined, which were input into a multivariate Cox regression model. The formula of EVA1B-relevant genomic model was conducted as follows: risk score = $\Sigma_{k-1}^{n}{\text{ Exp}}_{i}*e^{\text{HR}}i$ , in which *n* indicated the number of characteristic EVA1B-relevant genes, Exp_i_ indicated the expression of characteristic genes, and *e*^HR^*i* indicated the regression coefficients of genes derived from the multivariate Cox regression analysis. In accordance with this formula, risk score of each CRC patient was calculated. Thereafter, CRC patients were clarified into high- and low-risk subpopulations following the mean value of risk score. The risk score distribution, survival state, and heatmap of the expression of characteristic EVA1B-relevant genes were drawn following patients’ risk score. Kaplan–Meier curves of overall survival (OS), disease-specific survival (DSS), and progression-free survival (PFS) were depicted between high- and low-risk subpopulations using survival and survminer packages, followed by log-rank test. Thereafter, receiver operating characteristic (ROC) curves of 1-, 3-, and 5-year survival were established utilizing survivalROC package, and area under the curve (AUC) was calculated to assess the discrimination (34). Additionally, prognostic value of the EVA1B-derived genomic model was externally verified in the GSE14333, GSE39582, and GSE87211 cohorts.

### Assessment of Drug Sensitivity

The Genomics of Drug Sensitivity in Cancer (GDSC) project (https://www.cancerrxgene.org/) offers drug sensitivity information of 138 anticancer agents across approximately 75,000 experiments in 700 cancer cell lines (35). The half-maximal inhibitory concentration (IC50) that represented drug response was estimated with pRRophetic package (36).

### Nomogram Establishment

Uni- and multivariate Cox regression models were established to evaluate the associations of clinical indicators (age, gender, stage, T, N, and M) and EVA1B-relevant risk score with CRC patients’ OS. ROC curves were conducted to evaluate the prognostic value of this nomogram. Additionally, calibration curves were utilized to assess the consistency between the actual and nomogram-predicted survival probabilities.

### Clinical Specimens and Data Source

Formalin-fixed, paraffin-embedded specimens, including primary carcinoma specimens (*n* = 131), corresponding noncancerous normal tissues (*n* = 19) and liver metastasis specimens (*n* = 19) for immunohistochemistry (IHC) were obtained from 131 CRC patients who underwent surgery between 2014 and 2015. CRC primary carcinomas were assessed according to the 8th edition of the American Joint Committee on Cancer (AJCC) staging system. CRC patient data and tissue samples were obtained from the Liaoning Cancer Hospital. Patients included in the current study did not receive preoperative radiotherapy or chemotherapy prior to the study. The current study (20210804GP) was approved by the Ethics Committee of the Liaoning Cancer Hospital, Shenyang, China.

### Immunohistochemistry

EVA1B expression levels in samples were determined by IHC. Tissue sections were incubated with EVA1B antibody (Manufacturer: Atlas Antibodies; Catalog number: HPA043537). The level of EVA1B expression was determined by counting the percentages of positively stained immunoreactive cells and evaluating cell staining intensity. EVA1B IHC staining was scored as “−” ~ “+” and “++” ~ “+++,” which represented negative and positive, respectively. All samples were reviewed by two independent, experienced pathologists in who were blinded to the identity of the samples.

### Statistical Analysis

Statistical analysis was implemented with R software (v3.4.1; https://www.r-project.org/) and its appropriate packages. Comparison between groups was conducted utilizing Student’s *t*- or Wilcoxon rank-sum test. Spearman correlation test was adopted to determine the interactions between variables. Chi-square test was performed to analyze the correlation between EVA1B and clinicopathologic characteristics. Kaplan–Meier curves and the log-rank test were used to compare the OS between EVA1B expression level. The prognostic ability of the predictors for OS was evaluated by ROC curves and the AUC values. Univariate and multivariate Cox regression analyses were utilized to evaluate the independent prognostic value of EVA1B regarding OS. *p*-value <0.05 was set as the threshold.

## Results

### Analysis of Expression, Genetic and Epigenetic Alterations, and Immunological Characteristics of EVA1B Across Pan-Cancer

**Figure 1:**
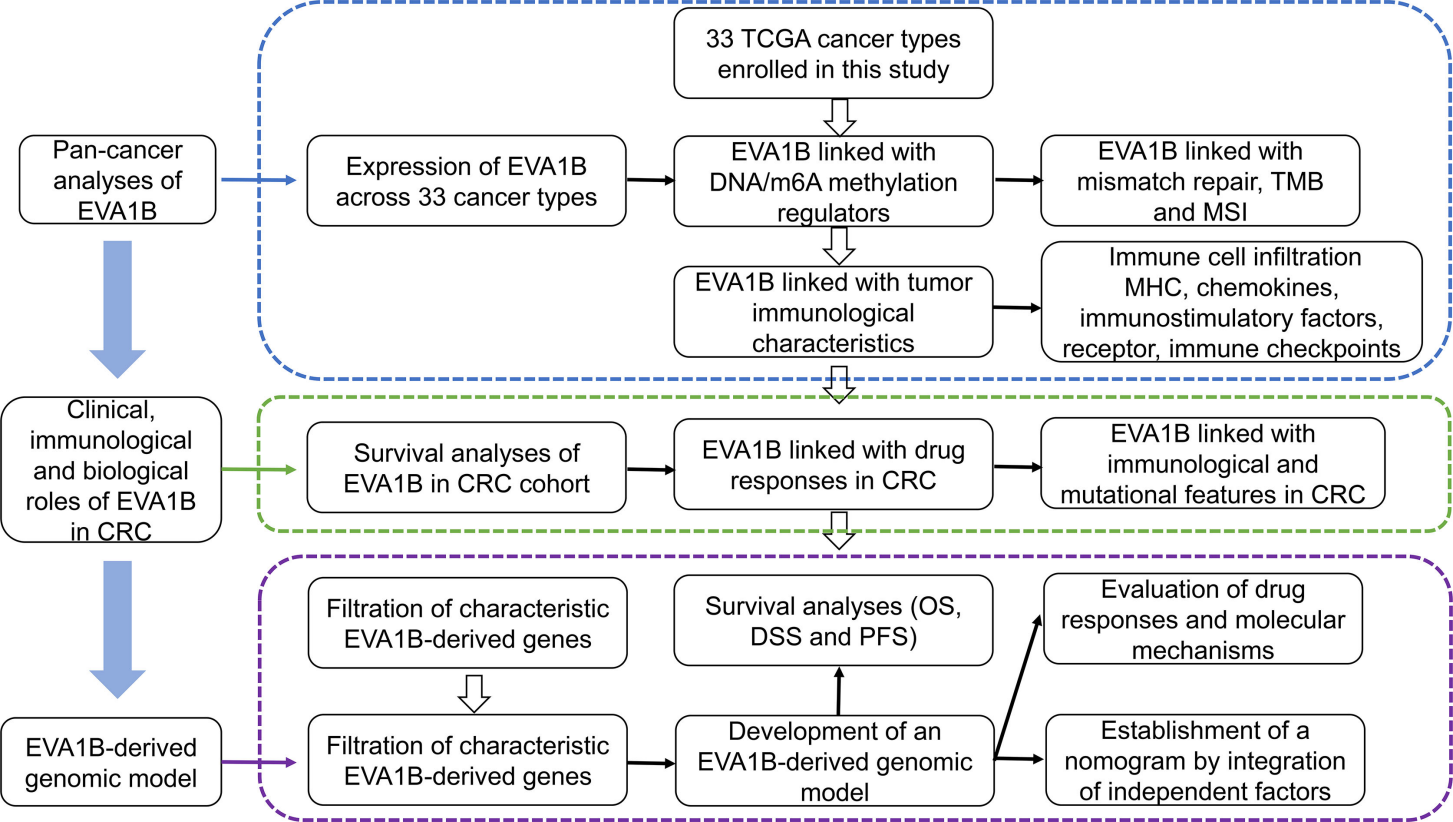
The workflow of this study.

**Figure 2:**
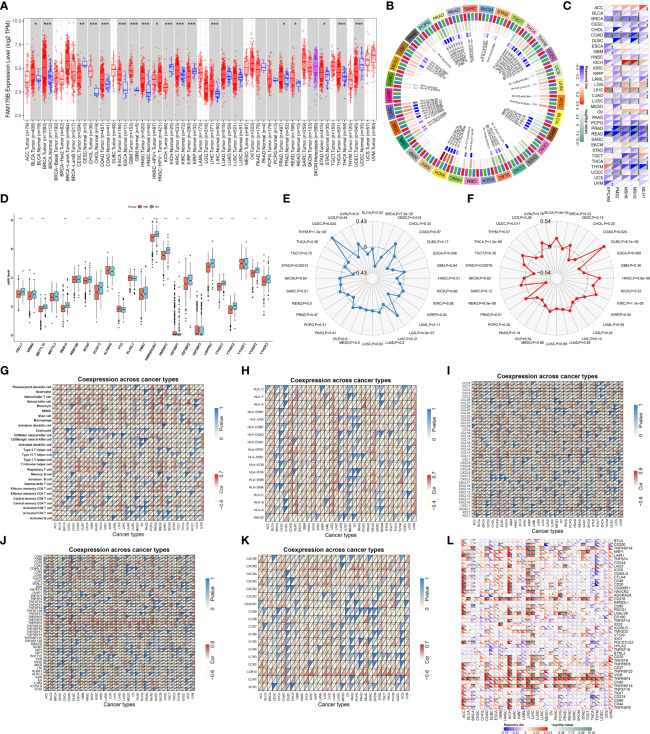
Analysis of expression, genetic and epigenetic alterations, and immunological characteristics of EVA1B across pan-cancer. **(A)** TIMER analysis identifies the difference in expression of EVA1B between diverse cancer types and matched normal specimens. **(B)** Circle diagram visualizes the interaction of EVA1B with four major DNA methyltransferases: DNMT1, DNMT2, DNMT3A, and DNMT3B across pan-cancer. The first outer ring, cancer types; the second ring, four DNA methyltransferases; the third ring, correlation coefficients; the fourth ring, *p*-values; and numbers in the inner ring, correlation coefficients and *p*-values. **(C)** Heatmap visualizes the relationship of EVA1B with five DNA mismatch repair genes: MLH1, MSH2, MSH6, PMS2, and EPCAM across diverse cancer types. For each relationship, the top left triangle is colored to indicate correlation coefficients, while the bottom right triangle is colored to indicate *p*-values. **(D)** Box plots depict the difference in expression of m^6^A regulators in high and low EVA1B expression groups across CRC specimens. **(E)** Radar chart shows the association of EVA1B with TMB in each cancer type. **(F)** Radar chart shows the association of EVA1B with MSI across pan-cancer. **(G–L)** Heatmaps visualize the association of EVA1B with **(G)** tumor-infiltrating immune cells, **(H)** MHC molecules, **(I)** chemokines, **(J)** immunostimulatory factors, **(K)** receptors, and **(L)** immune checkpoint molecules across cancer types. For each relationship, the top left triangle is colored to indicate *p*-values, while the bottom right triangle is colored to indicate correlation coefficients. ACC, adrenocortical carcinoma; BLCA, bladder urothelial carcinoma; BRCA, breast invasive carcinoma; CESC, cervical squamous cell carcinoma and endocervical adenocarcinoma; CHOL, cholangiocarcinoma; COAD, colon adenocarcinoma; DLBC, lymphoid neoplasm diffuse large B-cell lymphoma; ESCA, esophageal carcinoma; GBM, glioblastoma multiforme; HNSC, head and neck squamous cell carcinoma; KICH, kidney chromophobe; KIRC, kidney renal clear cell carcinoma; KIRP, kidney renal papillary cell carcinoma; LAML, acute myeloid leukemia; LGG, lower-grade glioma; LIHC, liver hepatocellular carcinoma; LUAD, lung adenocarcinoma; LUSC, lung squamous cell carcinoma; MESO, mesothelioma; OV, ovarian serous cystadenocarcinoma; PAAD, pancreatic adenocarcinoma; PCPG, pheochromocytoma and paraganglioma; PRAD, prostate adenocarcinoma; READ, rectum adenocarcinoma; SARC, sarcoma; SKCM, skin cutaneous melanoma; STAD, stomach adenocarcinoma; TGCT, testicular germ cell tumors; THCA, thyroid carcinoma; THYM, thymoma; UCEC, uterine corpus endometrial carcinoma; UCS, uterine carcinosarcoma; UVM, uveal melanoma. (^*^p < 0.05; ^**^p < 0.01; ^***^p < 0.001).

**Figure 1** depicts the workflow of this study. EVA1B, as known as FAM176B, displayed remarkably increased expression in most cancer types in comparison with corresponding normal tissues, containing BLCA, BRCA, CHOL, COAD, ESCA, GBM, HNSC, KIRC, LIHC, PRAD, READ, STAD, and THCA (**Figure 2A**). Differently, downregulated EVA1B was investigated in CESC, KICH, KIRP, and UCEC. DNA methyltransferases exert critical roles in changing chromatin structure and gene expression. We noted that EVA1B presented negative interactions with four major DNA methyltransferases containing DNMT1, DNMT2, DNMT3A, and DNMT3B in most cancer types (**Figure 2B**). Additionally, EVA1B was negatively correlated to DNA mismatch repair genes (MLH1, MSH2, MSH6, PMS2, and EPCAM) across cancer types (**Figure 2C**). M^6^A methylation represents the most common RNA modification, which affects the complexity of cancer progression (37). Especially, we focused on the interactions of EVA1B with m^6^A regulators in CRC. With the mean value of EVA1B expression, CRC patients were clustered into high and low EVA1B expression subpopulations. As depicted in **Figure 2D**, downregulated EVA1B was correlated to most m^6^A regulators like CBLL1, VIRMA, METTL14, and RBM15. TMB becomes a reliable biomarker of sensitivity to immune checkpoint blockage (38). In **Figure 2E**, EVA1B presented remarkably positive interaction with TBM across THYM, and LGG. Oppositely, it was negatively correlated with TMB across BRCA, CESC, ESCA, HNSC, STAD, and UCEC. MSI is a hypermutation phenotype caused by frequent polymorphisms in short repetitive DNA sequence as well as single nucleotide substitution due to DNA MMR defects (39). We noted the positive interaction of EVA1B with MSI in THCA, BLCA, DLBC, HNSC, KIRC, and PRAD but negative interaction with MSI in COAD, READ, STAD, and UCEC (**Figure 2F**). Thereafter, we analyzed the immunological characteristics of EVA1B across pan-cancer. Our data suggested that EVA1B was remarkably correlated to immune cell infiltration (**Figure 2G**), MCH molecules (**Figure 2H**), chemokines (**Figure 2I**), immunostimulatory factors (**Figure 2J**), receptors (**Figure 2K**), and immune checkpoint molecules (**Figure 2L**) across pan-cancer.

### Prognostic Significance of EVA1B and Its Association With Drug Sensitivity in CRC

**Figure 3:**
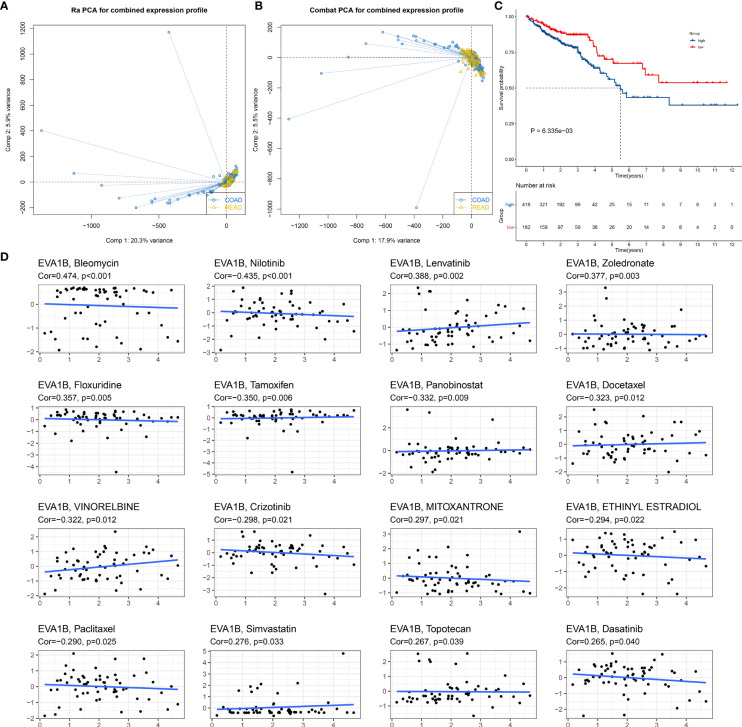
Prognostic significance of EVA1B and its association with drug sensitivity in CRC. **(A, B)** PCA diagrams depict the combined COAD and READ datasets from TCGA project before and after removal of batch effects. **(C)** Kaplan–Meier curves of OS for high and low EVA1B expression subpopulations. **(D)** Scatter plots show the correlation of EVA1B expression with drug sensitivity in CRC.

We also focused on the biological role and significance of EVA1B in CRC. We combined the COAD and READ datasets from TCGA project and removed batch effects (**Figures 3A, B**). Survival analysis was indicative that patients with high EVA1B expression possessed more undesirable OS outcomes than those with low EVA1B expression (**Figure 3C**). Targeted therapies play a key role in CRC management, and genetic alterations that attribute to CRC heterogeneity is correlated to the responses to targeted therapies (40). Reliable predictive biomarkers for targeted therapies remain greatly lacking. Thus, we evaluated whether EVA1B expression was correlated to drug sensitivity. As a result, EVA1B presented positive association with sensitivity to bleomycin, lenvatinib, zoledronate, floxuridine, mitoxantrone, simvastatin, topotecan, and dasatinib (**Figure 3D**). Differently, EVA1B expression was negatively correlated to sensitivity to nilotinib, tamoxifen, panobinostat, docetaxel, vinorelbine, crizotinib, ethinyl estradiol, and paclitaxel in CRC. Therefore, EVA1B might be a predictive marker of above agents.

### Immunological and Biological Significance of EVA1B in CRC

**Figure 4:**
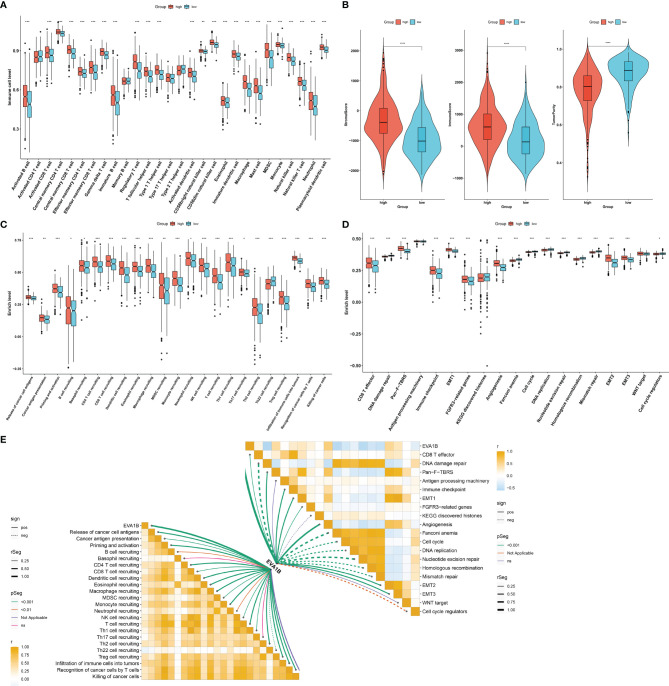
Immunological and biological significance of EVA1B in CRC. **(A)** Comparison of the infiltration of immune cells between high and low EVA1B expression subpopulations. **(B)** Evaluation of the difference in stromal score, immune score, and tumor purity in high and low EVA1B expression subpopulations. **(C)** Quantification of the activities of cancer immunity cycle in two subpopulations. **(D)** Comparing the activation of known biological signatures in two subpopulations. **(E)** Associations of EVA1B expression with the activities of cancer immunity cycle and known biological signatures across CRC specimens. (^*^p < 0.05; ^**^p < 0.01; ^***^p < 0.001).

Our ssGSEA results demonstrated the remarkably increased infiltration levels of most immune cells in high EVA1B expression subpopulation (**Figure 4A**). Additionally, high EVA1B expression was correlated to increased stromal and immune score as well as reduced tumor purity in CRC (**Figure 4B**). There were prominently enhanced activities of most steps within cancer immunity cycle in high EVA1B expression subpopulation (**Figure 4C**). We also investigated the increased activities of immune activation processes (like CD8+ T effector, and immune checkpoint) and stromal activation processes (like EMT, pan-F-TBRS, FGFR3-related genes, and angiogenesis) in high EVA1B expression subpopulation (**Figure 4D**). Additionally, the interactions of EVA1B expression with cancer immunity cycle and known biological processes were evaluated across CRC specimens. In **Figure 4E**, EVA1B expression presented positive correlations to most steps within cancer immunity cycle as well as immune and stromal activation processes.

### Association of EVA1B With Mutational Landscape in CRC

**Figure 5:**
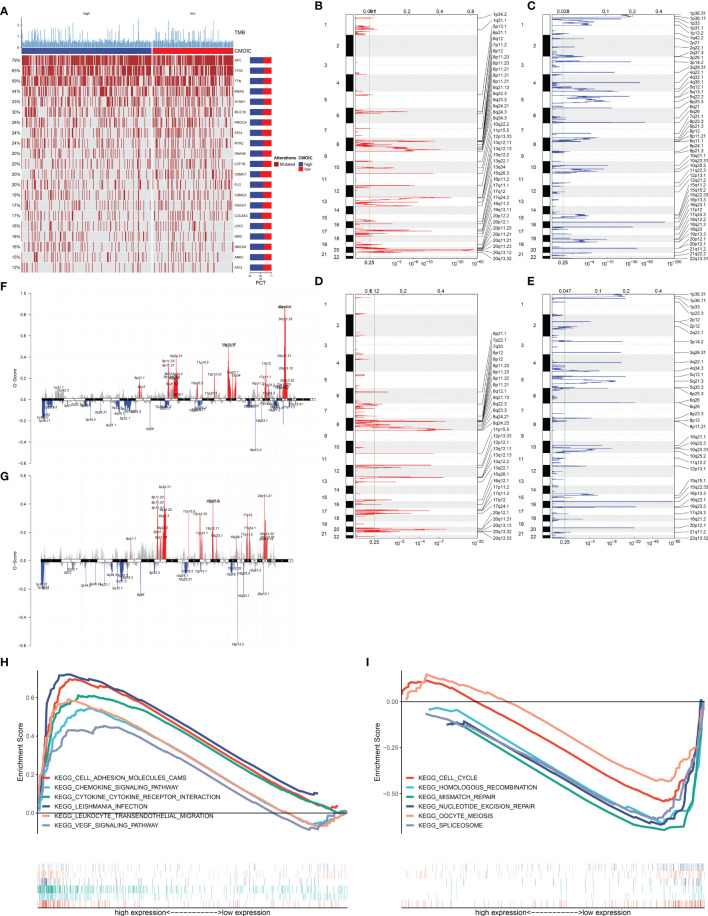
Association of EVA1B with mutational landscape and signaling pathways in CRC individuals. **(A)** Landscape of somatic mutation in high and low EVA1B expression CRC subpopulations. Genes are ranked following the mutational frequency. Upper portion of the chart displays TMB score of each patient. **(B, C)** Landscape of **(B)** amplification and **(C)** deletion in high EVA1B expression subpopulation. **(D, E)** Landscape of **(D)** amplification and **(E)** deletion in low EVA1B expression subpopulation. The genome is oriented vertically from top to bottom; GISTIC2.0 *q*-value at each locus is depicted from left to right. The green line indicates the cutoff value of *q*-value = 0.25. **(F, G)** Detection and comparison of amplification and deletion of copy number in high and low EVA1B expression subpopulations. **(H)** GSEA for the signaling pathways activated in high EVA1B expression subpopulation. **(I)** GSEA for the signaling pathways activated in low EVA1B expression subpopulation.

In **Figure 5A**, we evaluated the prevalence of somatic mutation in high and low EVA1B expression subpopulations. APC, TP53, and TTN ranked the first three mutational genes. Nevertheless, no prominent difference in somatic mutation was investigated in high and low EVA1B expression subpopulations. The GISTIC2.0 results demonstrated that amplification and deletion displayed higher frequencies in high EVA1B expression subpopulation (**Figures 5B, C**) compared with low EVA1B expression subpopulation (**Figures 5D, E**). Additionally, we calculated the G-score in accordance with the amplitude of the aberrations and the frequency of their incidence in CRC specimens. Following comparison, mutations occurred in more regions in high EVA1B expression subpopulation (**Figures 5F, G**).

### Signaling Pathways Associated With EVA1B

We further analyzed the signaling pathways involving EVA1B *via* GSEA. In **Figure 5H**, high EVA1B expression was positively correlated to cell adhesion molecules (CAMs), chemokine signaling pathways, cytokine–cytokine receptor interaction, Leishmania interaction, leukocyte trans-endothelial migration, and VEGF signaling pathways. Meanwhile, low EVA1B expression was negatively correlated to cell cycle, homologous recombination, mismatch repair, nucleotide excision repair, oocyte meiosis, and spliceosome (**Figure 5I**). The above data showed that EVA1B was involved in several oncogenic pathways, indicating the oncogenic role of EVA1B.

### Identification of EVA1B-Derived Genes and Their Biological Significance

**Figure 6:**
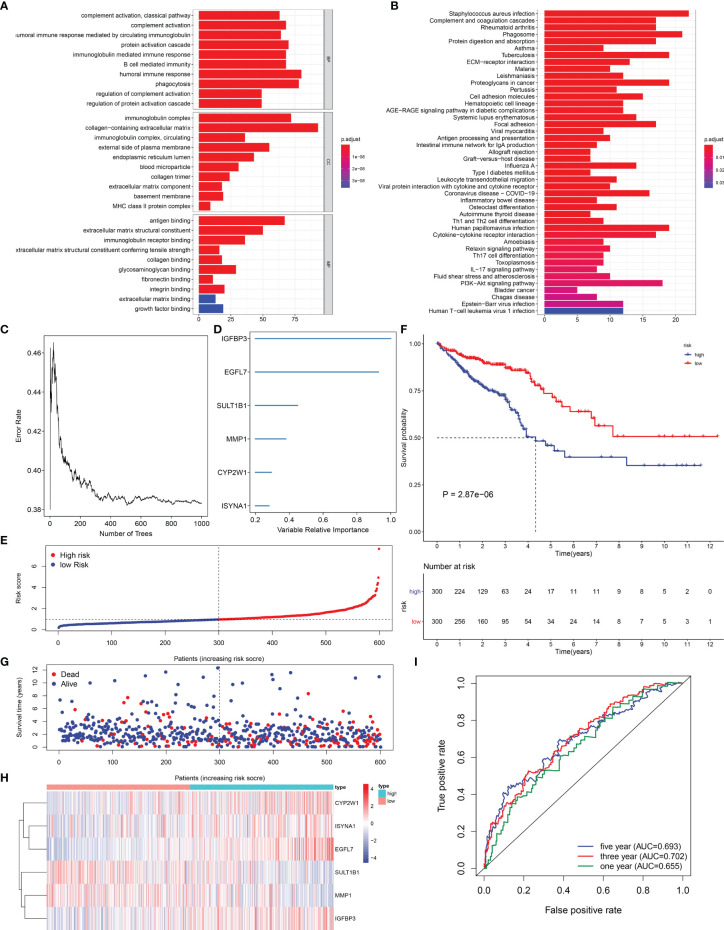
Exploitation of an EVA1B-derived genomic model for CRC prognosis. **(A, B)** GO and KEGG enrichment analyses of EVA1B-derived genes. **(C)** Random survival forest analysis for calculating the relative importance of EVA1B-derived genes. **(D)** Determination of characteristic EVA1B-derived genes with relative importance >0.28. **(E)** Distribution of high- and low-risk CRC subpopulations with the mean value (vertical dashed line) of EVA1B-derived risk score. **(F)** Kaplan–Meier curves of OS for high and low CRC subpopulations. **(G)** Distribution of survival status in two CRC subpopulations. **(H)** Heatmap for the expression of IGFBP3, EGFL7, SULT1B1, MMP1, CYP2W1, as well as ISYNA1 in two CRC subpopulations. **(I)** ROC curves at 1-, 3-, and 5-year OS outcomes in accordance with EVA1B-derived risk score.

Through the cutoffs of |fold-change| >1.5 and adjusted *p*-value <0.01, we determined 602 EVA1B-derived genes in CRC individuals (**Supplementary Table S1**). Their biological significance was further investigated. In **Figure 6A**, EVA1B-derived genes were mainly correlated to immune response in accordance with GO annotation results. Additionally, there were remarkable interactions of EVA1B-derived genes with tumorigenic pathways (such as PI3K-Akt signaling pathway, proteoglycans in cancer, and ECM-receptor interaction) and immune activation pathways (such as IL-17 signaling pathway, Th17-cell differentiation, cytokine–cytokine receptor interaction, Th1- and Th2-cell differentiation, antigen processing and presentation, intestinal immune network for IgA production, complement and coagulation cascades; **Figure 6B**). Overall, EVA1B-derived genes might exert remarkable roles in CRC progression.

### The EVA1B-Derived Genomic Model for CRC Prognosis

In total, 66 EVA1B-derived genes were prominently correlated to CRC patients’ prognosis utilizing univariate-Cox regression models (**Table 1**). With random survival forest analysis, we ranked the relative importance of above prognostic EVA1B-derived genes (**Figure 6C**). In accordance with relative importance >0.28, six characteristic EVA1B-derived genes were determined, containing IGFBP3, EGFL7, SULT1B1, MMP1, CYP2W1, as well as ISYNA1 (**Figure 6D**). On the basis of them, a multivariate-Cox regression model was conducted and risk scoring system was developed following the formula: risk score = (−0.117800092) * ISYNA1 expression + 0.067856101 * CYP2W1 expression + (−0.145078003) * MMP1 expression + (−0.107319254) * SULT1B1 expression + 0.427836868 * EGFL7 expression + 0.188926758 * IGFBP3 expression. With the mean value, we classified CRC patients into high- and low-risk subpopulations (**Figure 6E**). Survival analysis uncovered that low-risk CRC individuals displayed a remarkable survival advantage (**Figure 6F**). Nevertheless, no distinct difference in survival status was noted between high- and low-risk subpopulations (**Figure 6G**). Additionally, the heterogeneity in expression of IGFBP3, EGFL7, SULT1B1, MMP1, CYP2W1, as well as ISYNA1 was displayed in two subpopulations (**Figure 6H**). ROC curves confirmed that the EVA1B-derived genomic model possessed the well potency in estimating 1-, 3-, and 5-year OS probabilities (**Figure 6I**).

**Table 1:** Univariate-Cox regression models determine prognostic EVA1B-derived genes in CRC.

Gene	HR	Lower 95% CI	Upper 95% CI	*p*-value	Gene	HR	Lower 95% CI	Upper 95% CI	*p*-value
EVA1B	1.2498	1.0165	1.5367	0.0344	ADAM8	1.3206	1.0966	1.5904	0.0034
RCN3	1.2516	1.0360	1.5122	0.0200	SFRP2	1.1005	1.0178	1.1900	0.0163
UBTD1	1.4292	1.1012	1.8549	0.0073	GPX3	1.2600	1.0864	1.4613	0.0022
C11orf96	1.2038	1.0175	1.4243	0.0306	SCX	1.2693	1.0257	1.5707	0.0283
SIPA1	1.5314	1.1049	2.1226	0.0105	CAVIN1	1.2125	1.0151	1.4484	0.0336
CRIP2	1.2970	1.0562	1.5927	0.0131	RGCC	1.2466	1.0301	1.5085	0.0235
LTBP3	1.3106	1.0673	1.6093	0.0098	ISYNA1	1.2025	1.0062	1.4372	0.0426
CERCAM	1.1983	1.0009	1.4346	0.0489	ZNF385A	1.3667	1.1269	1.6574	0.0015
APOE	1.1119	1.0025	1.2333	0.0448	TPM2	1.2088	1.0411	1.4034	0.0128
RAB3IL1	1.3854	1.0918	1.7580	0.0073	SPOCK1	1.1938	1.0173	1.4010	0.0300
FSTL3	1.3310	1.1200	1.5818	0.0012	G0S2	1.1966	1.045	1.3700	0.0094
RGS19	1.3237	1.0551	1.6609	0.0154	GAS1	1.1596	1.0018	1.3423	0.0472
IER5L	1.3062	1.0439	1.6344	0.0195	MGP	1.1437	1.0137	1.2902	0.0291
HSD17B14	1.3673	1.0590	1.7655	0.0164	CRYAB	1.2665	1.0729	1.4951	0.0053
BGN	1.1667	1.0226	1.3312	0.0219	CNN1	1.1161	1.0099	1.2333	0.0312
GJA4	1.2785	1.0061	1.6246	0.0445	IGFBP3	1.2996	1.082	1.5611	0.0051
EGFL7	1.4972	1.2150	1.8449	0.0002	SCT	1.1864	1.0233	1.3754	0.0235
RTL8B	1.3178	1.0243	1.6954	0.0318	DEPP1	1.1990	1.0193	1.4103	0.0284
NOTCH3	1.2565	1.0292	1.5341	0.0249	CCL11	0.8402	0.7174	0.9841	0.0309
MYL9	1.1557	1.0126	1.3190	0.0319	ARL4C	1.2237	1.0249	1.461	0.0256
TIMP1	1.5239	1.2290	1.8895	0.0001	LY6E	1.1578	1.0031	1.3363	0.0452
PRRX2	1.2312	1.0121	1.4977	0.0375	AOC3	1.2133	1.0483	1.4043	0.0095
GPC1	1.3725	1.1201	1.6817	0.0023	RAMP1	1.1366	1.0276	1.2571	0.0128
PPP1R14A	1.2016	1.0010	1.4424	0.0487	TNS1	1.1874	1.0242	1.3766	0.0228
HOMER3	1.5016	1.1820	1.9078	0.0009	SPP1	1.0872	1.0002	1.1817	0.0495
YPEL3	1.3306	1.0722	1.6512	0.0095	SULT1B1	0.8293	0.7173	0.9588	0.0115
SERPING1	1.1556	1.0055	1.3281	0.0417	PRELP	1.2236	1.0686	1.401	0.0035
CHPF	1.3780	1.0874	1.7461	0.0080	RTL8A	1.1600	1.0017	1.3435	0.0475
FHL3	1.3222	1.0481	1.6679	0.0184	IGLV7-43	0.8716	0.7825	0.9708	0.0124
VSIG4	1.1643	1.0062	1.3472	0.0410	MMP1	0.8915	0.8164	0.9736	0.0106
COMP	1.1434	1.0333	1.2651	0.0095	RNU4-2	0.8745	0.7652	0.9995	0.0492
RTL8C	1.3160	1.0979	1.5774	0.0030	CYP2W1	1.0957	1.0049	1.1947	0.0384
IRF7	1.3400	1.0801	1.6623	0.0078	RN7SL2	0.9108	0.8295	1.0000	0.0500

### Verification of Prognostic Significance of EVA1B-Derived Genomic Model in CRC

**Figure 7:**
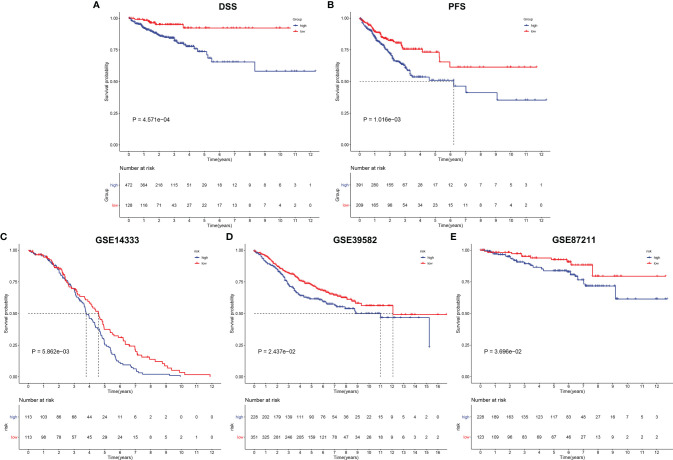
Verification of prognostic significance of EVA1B-derived genomic model in CRC. **(A, B)** Kaplan–Meier curves of DSS and PFS for high and low CRC subpopulations in TCGA cohort. **(C–E)** Kaplan-Meier curves of OS for high and low CRC subpopulations in the **(C)** GSE14333, **(D)** GSE39582, and **(E)** GSE87211 datasets.

Except for OS, our analysis demonstrated that high-risk subpopulation was indicative of more undesirable DSS and PFS outcomes in comparison with low-risk subpopulation in TCGA cohort (**Figures 7A, B**). The prognostic significance of the EVA1B-derived genomic model was verified in external cohorts. Our data confirmed that high-risk score was predictive of unfavorable OS outcome in the GSE14333, GSE39582, and GSE87211 datasets (**Figures 7C–E**).

### The EVA1B-Derived Genomic Model Predicts Drug Responses of CRC Patients

**Figure 8:**
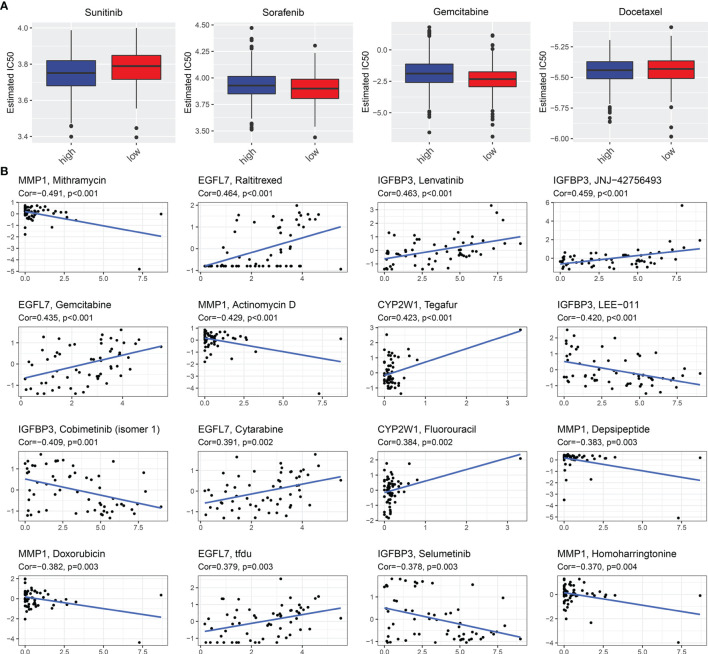
EVA1B-derived genomic model predicts drug responses of CRC patients. **(A)** Comparison of the estimated IC50 values of sunitinib, sorafenib, gemcitabine, and docetaxel between high- and low-risk CRC subpopulations. **(B)** Associations of EVA1B-relevant genes with IC50 values of small molecule compounds in CRC.

Further analysis was conducted to uncover whether this EVA1B-derived genomic model was predictive of drug responses of CRC individuals. In **Figure 8A**, we noted that high-risk group had significantly lower estimated IC50 of sunitinib and docetaxel relative to low-risk group, indicating that high-risk subpopulations presented higher sensitivity to sunitinib and docetaxel. Meanwhile, the low-risk group showed significantly lower estimated IC50 of sorafenib and gemcitabine, indicating that low-risk subpopulations were more likely to respond to sorafenib and gemcitabine. We also evaluated the interactions of EVA1B-derived genes with drug responses. As a result, MMP1 was negatively correlated to IC50 of mithramycin, actinomycin D, depsipeptide, and homoharringtonine; EGFL7 presented positive correlation to IC50 of raltitrexed, gemcitabine, cytarabine, and TFdU; IGFBP3 was positively correlated to IC50 of lenvatinib and JNJ-42756493 but negatively correlated to IC50 of LEE-011, cobimetinib (isomer 1), and selumetinib; CYP2W1 displayed positive relationship to IC50 of tegafur and fluorouracil (**Figure 8B**). These data indicated that EVA1B-derived genes might be correlated to sensitivity to above agents.

### Establishment of a Reliable Nomogram for Prediction of CRC Prognosis

**Figure 9:**
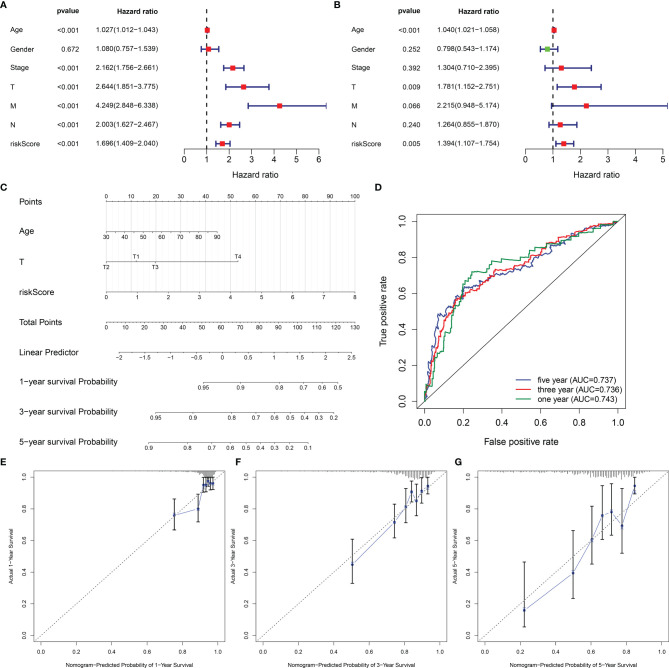
Establishment of a reliable nomogram for prediction of CRC prognosis. **(A, B)** Uni- and multivariate Cox regression models are conducted to uncover the association of clinical features and EVA1B-derived risk score with CRC survival outcome. **(C)** A prognostic nomogram is exploited through integrating independent prognostic indicators (age, T stage, and EVA1B-derived risk score) to estimate 1-, 3-, and 5-year survival probability. **(D)** Predictive efficacy of this nomogram is verified through ROC curves at 1-, 3-, and 5-year survival. **(E–G)** Calibration plots show the association of predicted 1-, 3-, and 5-year OS with actual survival duration.

Univariate-Cox regression analysis uncovered the remarkable association of age, stage, T, N, M, and EVA1B-derived risk score with CRC prognosis (**Figure 9A**). Among them, age, T, and EVA1B-derived risk score served as independent prognostic indicators of CRC (**Figure 9B**). By integrating above three independent prognostic indicators, this study exploited a nomogram to estimate CRC patients’ survival outcomes (**Figure 9C**). This EVA1B-derived risk score occupied the most contribution to the prediction of 1-, 3-, and 5-year OS duration. ROC curves confirmed the desirable efficacy in predicting patients’ survival outcomes (**Figure 9D**). Additionally, we evaluated the prediction performance of the nomogram with calibration curves. Our data demonstrated that 1-, 3-, and 5-year predicted by this nomogram was close to the actual survival duration (**Figures 9E–G**). Above data indicated the superior predictive capacity of this nomogram.

### Molecular Mechanisms Involving the EVA1B-Derived Genomic Signature

**Figure 10:**
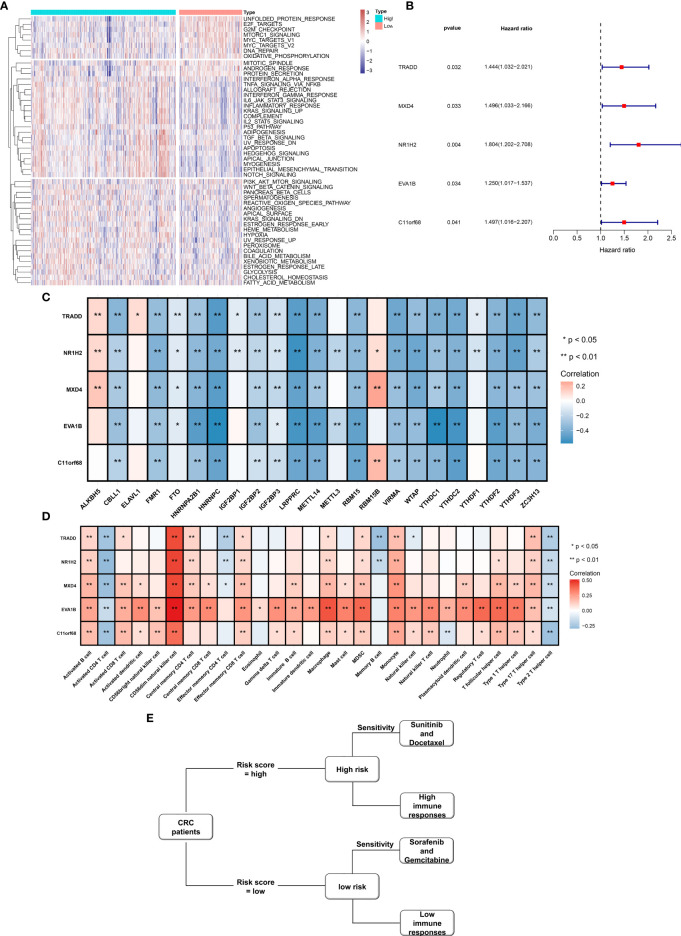
Molecular mechanisms involving EVA1B-derived genomic signature as well as prognostic value of EVA1B-derived genes and their interaction with m^6^A regulators and immune cell infiltration in CRC. **(A)** Heatmap visualizes the activities of hallmark gene signatures in high- and low-risk subpopulations. **(B)** Forest diagram depicts the interaction of EVA1B-derived genes with CRC clinical outcomes in accordance with univariate-Cox regression models. **(C)** Heatmap depicts the association of EVA1B-derived genes with m^6^A regulators across CRC specimens. **(D)** Heatmap visualizes the interaction of EVA1B-derived genes with immune cell infiltration in CRC. **(E)** Association among drug sensitivity, predictive immune responses, and risk status of CRC individuals. (^*^p < 0.05; ^**^p < 0.01).

We further investigated the molecular mechanisms involving the EVA1B-derived genomic signature. As depicted in **Figure 10A**, unfolded protein response, E2F targets, G2M checkpoint, mTORC1 signaling, MYC targets, DNA repair, and oxidative phosphorylation presented higher activities in low-risk subpopulation. Meanwhile, tumorigenic pathways were remarkably activated in high-risk subpopulation, such as P53 pathway, TGF-beta signaling, apoptosis, Hedgehog signaling, EMT, Notch signaling, and angiogenesis, indicative of high-risk subpopulation’s unfavorable survival outcomes. Moreover, immune activation pathways such as allograft rejection, inflammatory response, and complement were significantly activated in high-risk subpopulation.

### Prognostic Significance of EVA1B-Derived Genes and Their Interaction With m^6^A Methylation and Immune Cell Infiltration

**Figure 10B** demonstrates that TRADD, MXD4, NR1H2, EVA1B, and C11orf68 acted as risk factors of CRC outcomes. Additionally, we investigated that above EVA1B-derived genes presented positive correlations to m^6^A regulator ALKBH5 and RBM15B but negative correlations to other m^6^A regulators in CRC (**Figure 10C**). In **Figure 10D**, these EVA1B-derived genes were negatively correlated to activated CD4+ T cell, effector memory CD4+ T cell, memory B cell, and type 2 T helper cell but positively correlated to other tumor-infiltrating immune cells in CRC. **Figure 10E** shows the association among drug sensitivity, predictive immune responses, and risk status of CRC individuals.

### EVA1B Overexpression Is Correlated With CRC Tumorigenesis and Poor Outcomes in CRC Patients

**Figure 11:**
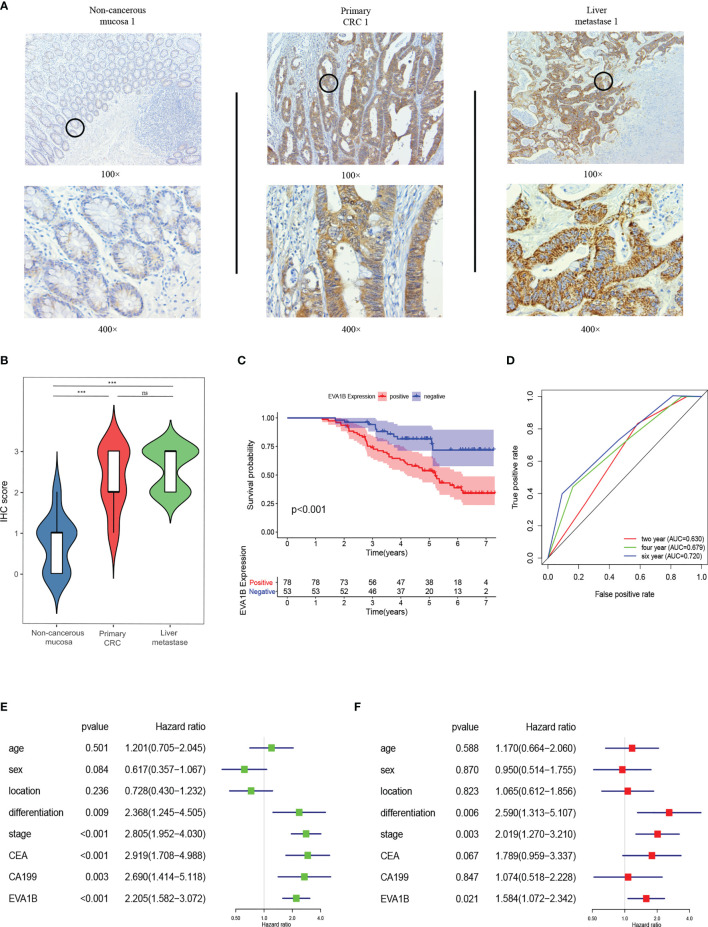
EVA1B overexpression is correlated with CRC tumorigenesis and poor outcomes in CRC patients. **(A, B)** Comparison of EVA1B expression in noncancerous mucosa, paired primary CRC, and liver metastases by IHC. **(C)** Kaplan–Meier plots of CRC specimens with negative and positive EVA1B expression. **(D)** Time-dependent ROC analysis of EVA1B showing the overall survival of patients with CRC. **(E, F)** Univariate and multivariate Cox regression of EVA1B expression and clinical parameters in CRC. ***p < 0.001; ns, no significance.

EVA1B IHC was performed on 19 pairs of primary CRC, corresponding noncancerous and matching liver metastasis specimens from 19 liver metastasis CRC patients. The findings showed that EVA1B was overexpressed in primary CRC tissues compared with the corresponding noncancerous normal tissues (*p* < 0.01). In addition, expression level of EVA1B was similar in the liver metastases samples to the expression level in matching primary CRC tissues without statistical significance (**Figures 11A, B**).

In addition, the relationship between EVA1B expression and clinicopathologic characteristics of 131 patients with CRC was explored (**Table 2**). Expression of EVA1B was highly correlated with pN stage (*p* < 0.001), pM stage (*p* = 0.005), AJCC stage (*p* = 0.001), CEA (*p* = 0.003), and CA199 (*p* = 0.002). However, the finding showed no significant correlation between EVA1B expression level and age, gender, tumor location, differentiation, and pT stage of CRC patients (**Table 2**).

**Table 2:** EVA1B staining expression associations with clinicopathologic and survival characteristics in CRC patients.

Variable	No.	Negative	Positive	*p*-value
Clinicopathologic characteristics				
Age (years)				0.922
<60	60	24	36	
≥60	71	29	42	
Gender				0.091
Male	71	24	47	
Female	60	29	31	
Location				0.596
Colon	63	24	39	
Rectum	68	29	39	
Differentiation				0.884
Poor	18	7	11	
Well	113	46	67	
pT stage				0.141
T1–T3	92	41	51	
T4	39	12	27	
pN stage				<0.001
N0	60	38	22	
N1–N2	71	15	56	
pM stage				0.005
M0	109	50	59	
M1	22	3	19	
AJCC stage				0.001
I	17	12	5	
II	36	25	11	
III	56	13	43	
IV	22	3	19	
CEA				0.003
≤5.0 (negative)	79	40	39	
>5.0 (positive)	52	13	39	
CA199				0.002
≤37.0 (negative)	114	52	62	
>37.0 (positive)	17	1	16	
Survival characteristics				
1-year survival	131	53 (100%)	78 (100%)	NS
2-year survival	125	52 (98.1%)	73 (93.6%)	NS
3-year survival	108	50 (94.3%)	58 (74.4%)	0.048
4-year survival	94	44 (83.0%)	50 (64.1%)	0.018
5-year survival	87	44 (83.0%)	43 (55.1%)	<0.001
6-year survival	77	42 (79.3%)	35 (44.9%)	<0.001
7-year survival	75	42 (79.3%)	33 (42.3%)	<0.001

NS, no significance.

Kaplan–Meier curve was generated to explore the prognosis value of EVA1B in these 131 CRC patients. The findings showed that CRC patients with high expression levels of EVA1B presented with poor OS compared with those with low expression levels of EVA1B (*p* < 0.001, **Figure 11C**). AUC values for EVA1B expression (0.630, 0.679, and 0.720 for the 2-, 4-, and 6-year OS, respectively) showed that EVA1B accurately discriminated between the high- and low-expression groups (**Figure 11D**). Univariate and multivariate Cox analyses were performed, indicating that EVA1B was an independent as a prognostic factor by adjusting for stage, gender, location, differentiation, stage, CEA, and CA199 (**Figures 11E, F**).

## Discussion

This study presented an integrative analysis of molecular characteristics, oncogenic roles, and relevant immune and pharmacogenomic features of EVA1B across pan-cancer, especially CRC. Our data demonstrated the remarkable upregulation of EVA1B expression in most cancer types. Additionally, its upregulation was significantly correlated to DNA methyltransferases, DNA mismatch repair genes, m^6^A regulators, TMB, and MSI across pan-cancer. Tumorigenesis is a multistep process where normal cells acquire genetic and epigenetic alterations contributing to tumor initiation and progression (41, 42). The pathological mechanisms of CRC initiation and progression include chromosomal instability, high MSI and methylation, contributing to oncogene, tumor suppressor gene, and mismatch repair-relevant gene mutations (43). Our data were indicative of the critical role of EVA1B in rectal and colon carcinogenesis.

High EVA1B expression was indicative of undesirable CRC patients’ clinical outcomes. Nevertheless, we did not note the distinct difference in survival status between high- and low-risk subpopulations due to this retrospective cohort. The role of EVA1B in survival status of CRC should be investigated in a prospective cohort in our future studies. Previous research proposed the upregulation of EVA1B in glioma (11). At present, the therapeutic regimen is surgical resection plus chemotherapy in more advanced or inoperable patients (44). Nevertheless, immunotherapy to supplement curative and palliative therapy has been undergoing clinical trials (45). Our further analysis demonstrated that EVA1B upregulation was correlated to enhanced immune cell infiltration, increased stromal and immune activation, and elevated activities of cancer immunity cycle. Additionally, higher frequencies of amplification and deletion were noted in high EVA1B expression subpopulation. Our data were indicative that EVA1B might be utilized as a promising immunotherapeutic predictor.

We further determined 602 EVA1B-derived genes in CRC that were mainly correlated to immune response, tumorigenic pathways (such as PI3K-Akt signaling pathway, proteoglycans in cancer, and ECM-receptor interaction), and immune activation pathways (such as IL-17 signaling pathway, Th17 cell differentiation, cytokine-cytokine receptor interaction, Th1 and Th2 cell differentiation, antigen processing and presentation, intestinal immune network for IgA production, complement and coagulation cascades), indicative of their remarkable roles in CRC progression. Utilizing random survival forest analysis, we conducted an EVA1B-derived genomic model, composed of IGFBP3, EGFL7, SULT1B1, MMP1, CYP2W1, and ISYNA1. Following verification in diverse external datasets, the genomic model reliably and independently predicted patients’ prognosis and relapse. Additionally, it possessed the potential in estimating drug responses, like sunitinib, sorafenib, gemcitabine, and docetaxel. There was remarkable correlation of characteristic EVA1B-derived genes with responses to small molecular compounds. TRADD, MXD4, NR1H2, EVA1B, and C11orf68 served as remarkable risk factors of CRC outcomes. Previously, modulation of TRADD restores cellular homeostasis as well as alleviates apoptosis (46). It triggers tumor inhibition through regulation of ULF-dependent p19Arf ubiquitylation (47). CDK4/6 inhibitor upregulates MXD4 expression that negatively modulates MYC in CD8+ T cells (48). NR1H2 modulates cholesterol homeostasis within human cells, controlling fitness and function of activated T cells (49). Nevertheless, more experimental evidence should be conducted to uncover the functions of above EVA1B-relevant genes in CRC.

A nomogram acts as a powerful tool to quantify an individual’s risk in a clinical setting through integrating diverse risk factors (50). Herein, this study utilized the nomogram in prediction of 1-, 3-, and 5-year OS probabilities *via* incorporating age, T stage, as well as EVA1B-derived genomic model for CRC individuals. Each indicator was assigned a score on the basis of its contribution to survival risk. Thereafter, ROC curves demonstrated that the nomogram presented the favorable efficiency in predicting an individual patient’ OS outcome. Additionally, calibrated curves confirmed the actual survival duration agreed with the nomogram-estimated survival duration. A few limitations of our research will be pointed out. The functional role of EVA1B in tumor immunity requires in-depth experimental verification. Moreover, prognostic value of EVA1B needs to be verified in larger CRC cohorts.

## Conclusion

Overall, our study conducted an integrative analysis to uncover molecular characteristics, oncogenic roles, and relevant immune and pharmacogenomic features of EVA1B in CRC. Our findings indicated that EVA1B acted as a convincing prognostic marker as well as a predictor of therapeutic responses.

## Data Availability Statement

Publicly available datasets were analyzed in this study. These data can be found here: https://portal.gdc.cancer.gov/ and GEO, https://www.ncbi.nlm.nih.gov/geo/, under accession numbers GSE14333, GSE39582, and GSE87211.

## Ethics Statement

The studies involving human participants were reviewed and approved by the human ethics committees of the Liaoning Cancer Hospital. The patients/participants provided their written informed consent to participate in this study.

## Author Contributions

BM and YML conceived and designed this study. YML and KW performed the bioinformatics analyses and visualization. YL collected data and performed the statistical analysis. BM, YML and QM wrote the original draft. BM and YML revised the manuscripts. All authors revised and approved the final manuscript.

## Funding

This study was supported by the National Natural Science Foundation of China (81902383); the Revitalizing Liaoning Talents Program (XLYC1907004); the Young and Middle-aged Scientific and Technological Innovation Talent Support Plan of Shenyang City (RC200223); Cultivation Program of National Science Foundation of Liaoning Cancer Hospital (2021-ZLLH-03); Cultivation Program of National Science Foundation of Liaoning Cancer Hospital (2020-ZLLH-44); Natural Guiding Plan Foundation of Liaoning Province (2019-ZD-0584); and Beijing Xisike Clinical Oncology Research Foundation (Y-QL202101-0039).

## Conflict of Interest

The authors declare that the research was conducted in the absence of any commercial or financial relationships that could be construed as a potential conflict of interest.

## Publisher’s Note

All claims expressed in this article are solely those of the authors and do not necessarily represent those of their affiliated organizations, or those of the publisher, the editors and the reviewers. Any product that may be evaluated in this article, or claim that may be made by its manufacturer, is not guaranteed or endorsed by the publisher.

## Supplementary Material

Supplementary Table S1 The information of 602 EVA1B-derived genes in CRC patients.

## Abbreviations

CRC, colorectal cancer; IHC, immunohistochemistry; MSI, microsatellite instability; MSS, microsatellite-stable; TMB, tumor mutational burden; CNV, copy number variation; TCGA, The Cancer Genome Atlas; TIMER, Tumor Immune Estimation Resource; ssGSEA, single-sample gene set enrichment analysis; GSVA, gene set variation analysis; ESTIMATE, estimation of stromal and immune cells in malignant tumors using expression data; EMT, epithelial-mesenchymal transition; GO, Gene Ontology; KEGG, Kyoto Encyclopedia of Genes and Genomes; OS, overall survival; DSS, disease-specific survival; PFS, progression-free survival; ROC, receiver operating characteristic; AUC, area under the curve; GDSC, Genomics of Drug Sensitivity in Cancer; IC50, half-maximal inhibitory concentration.
